# Epithelial barrier function properties of the 16HBE14o- human bronchial epithelial cell culture model

**DOI:** 10.1042/BSR20201532

**Published:** 2020-10-16

**Authors:** Patrick J. Callaghan, Bryan Ferrick, Elizabeth Rybakovsky, Sunil Thomas, James M. Mullin

**Affiliations:** 1 Lankenau Institute for Medical Research, 100 Lancaster Avenue, Wynnewood, PA 19096, U.S.A.; 2 Department of Biomedical Engineering, Drexel University, Philadelphia, PA 19104, U.S.A.

**Keywords:** barrier function, bronchial, CFTR, tight junction, transepithelial electrical resistance, tumour necrosis factors

## Abstract

The human bronchial epithelial cell line, 16HBE14o- (16HBE), is widely used as a model for respiratory epithelial diseases and barrier function. During differentiation, transepithelial electrical resistance (TER) increased to approximately 800 Ohms × cm^2^, while ^14^C-d-mannitol flux rates (J_m_) simultaneously decreased. Tight junctions (TJs) were shown by diffusion potential studies to be anion-selective with P_C1_/P_Na_ = 1.9. Transepithelial leakiness could be induced by the phorbol ester, protein kinase C (PKC) activator, 12-*O*-tetradecanoylphorbol-13-acetate (TPA), and the proinflammatory cytokine, tumor necrosis factor-α (TNF-α). Basal barrier function could not be improved by the micronutrients, zinc, or quercetin. Of methodological significance, TER was observed to be more variable and to spontaneously, significantly decrease after initial barrier formation, whereas J_m_ did not significantly fluctuate or increase. Unlike the strong inverse relationship between TER and J_m_ during differentiation, differentiated cell layers manifested no relationship between TER and J_m_. There was also much greater variability for TER values compared with J_m_. Investigating the dependence of 16HBE TER on transcellular ion conductance, inhibition of the Cystic Fibrosis Transmembrane Conductance Regulator (CFTR) chloride channel with GlyH-101 produced a large decrease in short-circuit current (I_sc_) and a slight increase in TER, but no significant change in J_m_. A strong temperature dependence was observed not only for I_sc_, but also for TER. In summary, research utilizing 16HBE as a model in airway barrier function studies needs to be aware of the complexity of TER as a parameter of barrier function given the influence of CFTR-dependent transcellular conductance on TER.

## Introduction

Epithelial cell layers are the interface between the outside environment and the interior of the body, functioning as a physical barrier and preventing pathogens, allergens, and noxious compounds from entering interstitial tissue and blood vessels [1,2]. Proteins such as occludin and claudins form oligomeric complexes—known as tight junctions (TJs)—which create a continuous gasket-like seal around the apical perimeter of epithelial cells [2]. This seal plays a role in establishing apical-basal polarity and regulating the paracellular permeability of the cell layer [2,3].

An intact barrier is vital to the functioning of organ systems such as the gastrointestinal, renal, respiratory, and genitourinary tracts. Disruption of these barriers and consequential increased non-specific permeability of the epithelium can manifest in conditions like celiac disease, inflammatory bowel disease, and allergies [4–7]. Inflammatory conditions of both the upper and lower respiratory tracts, such as rhinitis and asthma, have been shown to be exacerbated by dust mite fecal proteases that induce paracellular leak across the epithelium and provide inhaled antigens access to underlying tissue [8–11]. Viruses often need to cross epithelial barriers in order to infect a host or be transmitted. Human immunodeficiency virus 1 (HIV-1) alters claudin expression in genital epithelial cells, allowing for the virus to disseminate into the lumen of the genitourinary tract and be transmitted to a new host [12]. HIV-1 down-regulates TJ protein expression and induces increased permeability of the blood–brain barrier and the gastrointestinal tract, initiating neuropathogenesis and gastrointestinal distress, respectively [12].

TJ proteins also allow for the creation of important electrochemical gradients that in turn allow for unidirectional movement of specific solutes across the cell layer, such as occurs in the nephrons of kidneys [2,13]. In the thick ascending limb of nephrons, claudin-16 and claudin-19 form a cation-selective paracellular channel required for magnesium reabsorption, with mutations in these genes causing hypomagnesemia [13]. It is evident that TJ proteins play a necessary role in the formation and maintenance of an epithelial barrier required for normal physiological functioning of many organ systems.

The bronchial airways form a route for transporting inhaled oxygen to the alveoli. The bronchial epithelial barrier prevents penetration of inhaled detrimental compounds and pathogens into the bloodstream [1]. The same pattern of claudin expression perturbation leading to pathologic conditions is observed in this epithelium like any other. For example, a claudin-18 deficiency has been associated with the development of asthma [14]. Altering claudin expression in airway epithelia is associated with several other pulmonary diseases as well: chronic obstructive pulmonary disease (COPD), acute respiratory distress syndrome (ARDS), and even lung cancer [15]. Given that the airway epithelium is constantly subjected to inhaled pathogens, allergens, and carcinogenic compounds, it is imperative for this barrier to be intact, thereby preventing a variety of respiratory diseases [1,2,15].

16HBE14o- (16HBE), a human bronchial epithelial cell line, was originally obtained from a 1-year-old male and immortalized with SV40 plasmid [16]. The expression of an apical chloride channel, the cystic fibrosis transmembrane conductance regulator (CFTR), in differentiated cell populations allows for 16HBE to serve as a model for studying cystic fibrosis [16–18]. This cell line also serves as an important model in the study of COPD, asthma, and lung cancer pathogenesis and treatment due to the fact that 16HBE retains many of the functions and morphology of differentiated normal bronchial epithelial cells [19–23]. While many studies of pulmonary barrier function exist for primary human bronchial epithelium and Calu-3—an additional immortalized bronchial epithelial cell line — less literature is available on 16HBE barrier function. Thus, the present study sought to characterize the general barrier properties of 16HBE cell layers as well as identify disease-relevant compounds that can alter 16HBE barrier function and TJ protein expression.

This is the first study to offer an explanation for the spontaneous decline of 16HBE TER after reaching its peak value, a phenomenon reported but not explained in prior publications utilizing 16HBE [23,24]. This decline is shown to be due not to compromised cell layer barrier function but rather to increased transcellular conductance involving in part the CFTR channel.

## Materials and methods

### Cell culture

The 16HBE cell culture was obtained from Millipore Sigma (St. Louis, MO) and used for 15 weeks before returning to frozen cell stocks. After reaching confluence, cells were trypsinized (0.25% trypsin and 2.21 mM EDTA) (Corning Cellgro, Manassas, VA) and then passaged on a weekly basis. Cells were seeded at 1.5 × 10^6^ cells per Falcon 75-cm^2^ culture flask with 25 ml Dulbecco’s Modified Minimum Essential Medium (Corning Cellgro, Manassas, VA), supplemented with 2 mM l-Glutamine (Corning Cellgro, Manassas, VA), 10% fetal bovine serum (Seradigm, VMR, Inc., Radnor, PA), 1% non-essential amino acids (Corning Cellgro, Manassas, VA), and 1 mM sodium pyruvate (Corning Cellgro, Manassas, VA). Cultures were incubated at 37°C in 95% air/5% CO_2_ atmosphere.

### Transepithelial permeability measurements

Cells were seeded into sterile Millicell polycarbonate (PCF) cell culture inserts (30 mm diameter with 0.4 μm pore size [EMD Millipore, Burlington, MA] on day 0 at a seeding density of 1.0 × 10^6^ or 2.0 × 10^6^ cells/insert. Four Millicell PCF inserts were placed in 100 mm Petri dishes. On day 1, all cell layers were refed with control medium containing 50 U/ml penicillin and 50 μg/ml streptomycin (2 ml apical, 15 ml basolateral). The same refeed procedure was performed on day 4. All treatments with tumor necrosis factor α (TNF-α), 12-*O*-tetradecanoylphorbol-13-acetate (TPA), zinc, and quercetin began on day 6 (when the cell layer was confluent).

Cell layers were refed with control medium or treatment medium on the morning of experiments and allowed to incubate at 37°C for 90 min prior to electrophysiological readings. Transepithelial potential difference was measured at 37°C using 1 M NaCl salt bridges in series with calomel electrodes. Transepithelial electrical resistance (TER) was measured at room temperature (RT) or at 34°C using 1 s, 40 μamp direct current pulses (through 1 M NaCl salt bridges in series with Ag/AgCl electrodes) in a custom-made Lexan chamber designed to hold the Millicell PCF inserts [25,26]. Ohm’s law was used to calculate TER: V = iR. Current-passing and voltage-measuring salt bridges were positioned above and below the center point of the cell layers.

Following TER measurements, the basal-lateral medium was aspirated and replaced with 15 ml of medium containing 0.1 mM, 0.1 μCi/ml ^14^C-d-mannitol (PerkinElmer, Boston, MA) and incubated at 37°C. Triplicate 50-μl samples were taken from the basolateral medium to determine the specific activity via liquid scintillation counting (LSC). Duplicate 250-μl samples were taken from the apical side at either 60 or 90 min for LSC to determine flux rates (^14^C-d-mannitol flux rate, J_m_). The flux rate (in picomoles/min/cm^2^) was calculated for ^14^C-d-mannitol diffusing across the cell layer.

### Light microscopy

16HBE confluent cell layers cultured in Millicell PCF cell inserts were rinsed three times in 4°C, 0.15 M NaCl then fixed for 30 min at RT in 4% formaldehyde in Phosphate Buffered Saline (PBS). Cell layers were then stored at 4°C in PBS. Cell layers still attached to polycarbonate filters were embedded in paraffin. Twenty-micron thickness sections were made perpendicular to the plane of the filter/cell layer using a Reichert–Jung microtome. Cross-sections were then stained with Hematoxylin and examined using a Zeiss Axioplan light microscope.

### Treatment with GlyH-101

Medium containing the CFTR channel inhibitor, GlyH-101 (EMD Millipore, Burlington, MA), was filter-sterilized with 0.2-μm disc filter units (Corning, Inc., Corning, NY) at a final concentration of either 5 or 50 μM. For physiological experiments, GlyH-101 was added to the apical compartment and measurements were taken at 15- and 30-min after exposure. Medium containing ouabain (Sigma–Aldrich, St. Louis, MO) at a concentration of 1 mM was applied to the basal-lateral cell surface.

### Treatment with TNF-α and TPA

Medium containing the cytokine, TNF-α (Peprotech, Inc., Rocky Hill, NJ), at a concentration of 133 ng/ml was applied to the apical and basal-lateral cell surfaces. Physiological measurements were taken at 5-, 24-, and 48 h after the initial exposure.

Medium containing the phorbol ester, TPA (Sigma–Aldrich, St. Louis, MO) (1 mM stock concentration in ethanol), at a concentration of 10^−7^ M was applied to the apical and basal-lateral cell surfaces. Physiologic measurements were taken at 5, 24, and 48 h after the initial exposure, with re-exposure to TPA at the 24-h time point.

### Treatment with zinc and quercetin

Zinc sulfate heptahydrate (Sigma–Aldrich, St. Louis, MO) was dissolved in deionized distilled water to make a 200 mM stock and then diluted to concentrations of 50, 100, or 200 μM in culture medium. The appropriate concentration was then added to both the apical and basal-lateral compartments. Physiological measurements were taken at 24 and 48 h after the initial exposure.

Quercetin-containing medium (Sigma–Aldrich, St. Louis, MO) was prepared by adding dry chemical to culture medium to a concentration of 400 μM. Dissolving quercetin directly into the culture medium required constant stirring for 75 min at a temperature of 37–40°C. This 400 μM solution was then serially diluted to make 200 and 100 μM Quercetin concentrations. The appropriate concentration was then added to both the apical and basal-lateral compartments. Physiological measurements were taken at 24 and 48 h after the initial exposure.

### Immunoblot analysis of TJ proteins

Cells were harvested from Millicell PCF inserts by the following method: cell layers were washed five times in cold PBS; 500 μl of lysis buffer with protease and phosphatase inhibitors was then added to each PCF. The cell layer was physically scraped off the filter; the suspension was collected, flash-frozen, and stored at −80°C. Once thawed, whole-cell lysates were prepared by sonication and ultracentrifugation. Samples of these lysates were analyzed by PAGE using a 4−20% gradient Tris-glycine gel (Invitrogen, a division of Thermo Fisher Scientific) at 120 V for 80 min. Precision Plus Kaleidoscope Protein Standards (Bio-Rad, Inc., Hercules, CA) were included on each gel. Proteins were transferred at 30 V for 1 h from the gel to a nitrocellulose membrane. The membranes were then washed three times with PBS-T (0.3% Tween 20) for 10 min and blocked with 5% milk/PBS-T at RT for 1 h. Membranes were incubated with the specific primary antibody (anti-claudin-1, -3, -4, -5, -7, - tricelluin, or -occludin [Thermo Fisher Scientific] and anti-claudin-2 [Abcam, Cambridge, MA]), at 0.5 μg/ml in 5% milk/PBS overnight at 4°C. The membranes were again washed three times, 10 min each with PBS-T, and then incubated with the secondary antibody (rabbit anti-mouse- or goat anti-rabbit-IgG antibody labeled with horseradish peroxidase [Southern Biotech, Birmingham, AL]) for 1 h at RT. The membranes were washed four times, 10 min each with PBS-T, and then treated for 10–60 s with Western Lightning Plus-ECL chemiluminescence reagents (PerkinElmer). The membranes’ band densities were quantified using the Bio-Rad ChemiDoc Imaging System. The band densities of the normalized experimentally treated cell samples were compared with normalized corresponding control-cell sample densities. Data were statistically analyzed using the paired Student’s *t* test. All data were expressed as the mean ± standard error of the mean.

### Diffusion potential measurements

Two solutions were prepared for this experiment: serum-supplemented, MOPS-buffered saline (Saline-A), and reduced NaC1, serum-supplemented, MOPS-buffered saline (Saline B).

Saline A: 137 mM NaC1, 4.6 mM KC1, 2.7 mM CaC1_2_, 1.5 mM MgSO_4_, 2.3 mM NaH_2_PO_4_, 3.5 mM NaHCO_3_, 5 mM glucose, 2 mM glutamine, 10% fetal bovine serum, and 9.2 mM Na-MOPS in deionized distilled (dd) water.

Saline B: Same as solution A except NaCl was only 37 mM; 176 mM mannitol was added to balance the osmolarity to equal that of Saline A. The measured pH of each solution was 7.3. Saline A’s osmolarity was 308 mOsM, while Saline B’s was 312 mOsM.

Cell layers were refed the morning of the experiment in control medium and baseline TER measurements were recorded 90 min later. Culture medium was replaced with Saline A on both sides of the cell layer and the potential difference across the cell layer was recorded 2, 5, 10 min later. The cell layers were moved to a new Petri dish where the fluid in the apical compartment was replaced with Saline B and the basolateral compartment received Saline A. This established a 100 mM NaCl gradient directed apically across the cell layer. Potential difference across the cell layer was recorded 2, 5, 10 min after saline exposure. The cell layers were then bathed with Saline A on both sides and potential difference TER measurements were recorded at RT.

The biological voltage that existed at t = 10 min with no NaCl gradient imposed across the cell layer was subtracted from the potential difference observed at t = 10 min with the 100 mM NaCl gradient imposed. Additionally, the liquid–junction potential—defined here as the voltage observed across a blank PCF with no NaCl gradient imposed—was subtracted as well. This varied from only 0.1–0.0 mV. The Goldman Equation was used to determine the relative permeability of 16HBE cell layers to chloride and sodium ions (P_Cl_/P_Na_) using the adjusted diffusion potential at t = 10 min.

$$\text{Goldman}\;\text{Equation:}\;\;\text{P}\text{.D}\text{.}=\frac{RT}{F}\text{ln}\left(\frac{P_{Na}\left[Na_{apical}\right]+P_{Cl}\left[Cl_{basolateral}\right]}{P_{Na}\left[Na_{basolateral}\right]+P_{Cl}\left[Cl_{apical}\right]}\right).$$

### Statistics

Determination of statistical significance in these studies was performed by means of two-sided Student’s *t* tests when comparing a single control group with a single experimental group, or one-way ANOVA when multiple groups with sufficient sample sizes were being compared. In both cases, significance was claimed when *P*<0.05.

## Results

### Morphology

**Figure 1:**
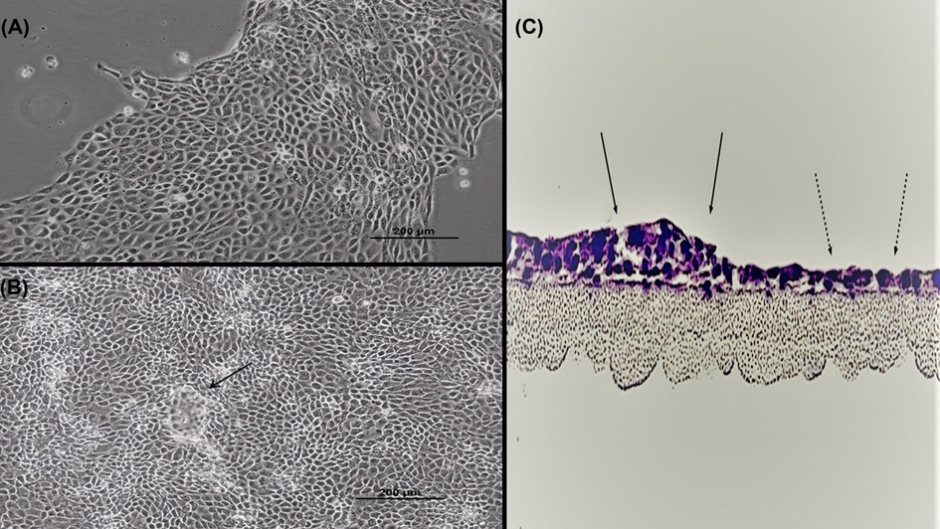
Morphology of 16HBE cell cultures Phase contrast image of a subconfluent (**A**) and 1 day post-confluent (**B**) cell layer grown in a Falcon T75 culture flask as described in ‘Materials and methods’ section (100×). A dome/hemicyst is pointed out by the black arrow. (**C**) A 16HBE confluent cell layer on a permeable polycarbonate filter was cross-sectioned and then stained with Hematoxylin and Eosin, showing that 16HBE can form both simple monolayer (dashed arrows) and multilayered (solid arrows) epithelial layers at confluence.

Phase contrast microscopy revealed that 16HBE cells in subconfluent cultures typically aggregate together in large islands of closely opposed cells, a characteristic observed in many types of epithelial cell cultures (Figure 1A). Once the cell layer reaches confluence, domes/hemicysts can occasionally be observed (Figure 1B). These hemicysts are pockets of fluid trapped between the cell layer and substratum; they indicate uniform polarity among cells, unidirectional salt and water transport, and strong barrier function as the cell layer is effectively ‘trapping’ under itself the electrolytes transported unidirectionally from the culture medium above the cell layer to the space between the cell layer and the culture dish [27,28]. However, the hemicysts were observed with much less frequency and smaller size compared with other epithelial cell lines like LLC-PK_1_, MDCK, and CACO-2. Cross-sectioning of a confluent cell layer revealed that most portions of the cell layer feature a monolayer, while a minority of regions are stratified (Figure 1C). It is unclear what contributes to 16HBE becoming stratified in certain portions—or whether these regions differ from the monolayer areas in terms of barrier properties.

### Transepithelial physiology

**Figure 2:**
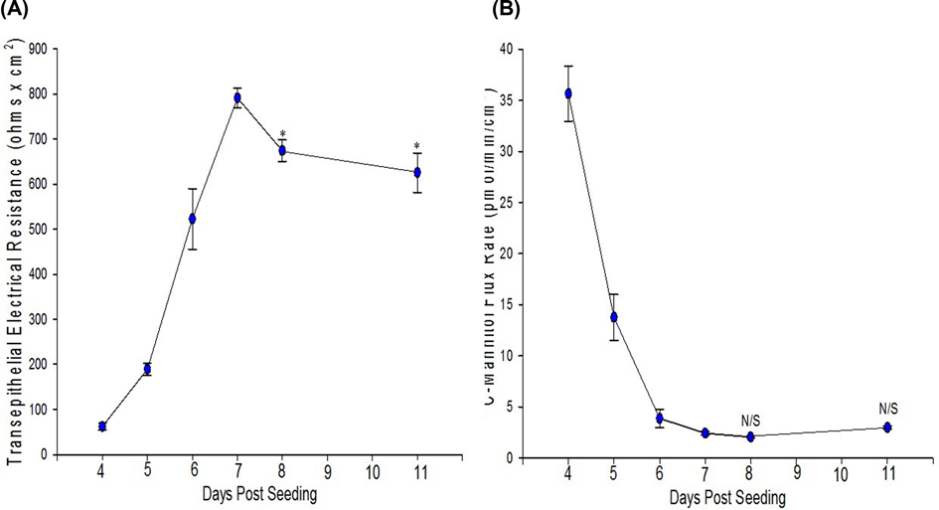
Time course of the development of 16HBE TER (A) and the ^14^C-mannitol flux rate (B) Seeded at 1.0 × 10^6^ cells per 4.2 cm^2^ PCF, TER, and J_m_ were measured as described in ‘Materials and methods’ section beginning on day 4 post-seeding. Data shown are mean ± standard error of mean. The data are composites of multiple time courses, *n*=6 cell layers for days 4, 5, and 6; *n*=7 for days 7 and 8; *n*=4 for day 11. * indicates *P*<0.05 when compared with day 7 values; N/S, not significant when compared with day 7 (Holm–Sidak method following one-way ANOVA).

Once a complete barrier is formed, 16HBE cell layers can function as relatively strong barriers evidenced by a mean TER of 713 Ω × cm^2^ and mean J_m_ of 2.1 pmol/min/cm^2^ across all the experiments used during this project (Table 1). It was typically observed that this barrier strength increased during the differentiation process, evidenced by a simultaneous early rise in TER and decline in J_m_ as a function of days post-seeding leading up to confluence and differentiation (Figure 2). By day 7, TER increases to values between 700 and 800 Ohms × cm^2^ before beginning to decrease (Figure 2A). J_m_ decreases over 11 days to values of 1.5–3.0 pmol/min/cm^2^ and does not significantly fluctuate beyond this range (Figure 2B). For well-differentiated cell layers with J_m_ values below 2.0 pmol/min/cm^2^, the relationship between TER and J_m_ was seemingly random. Ideally TER and J_m_ can both be used as valid indicators of a cell layer’s barrier strength albeit to different classes of compounds and charged vs. non-charged molecules, respectively. However, a wide range of TER values were observed for cell layers with a very narrow range of J_m_ values (Table 1). This combined with the time course showing the decrease in TER after reaching its peak (8 days post-seeding) without an accompanied change in J_m_ suggested perhaps that TER alone is not a useful indication of 16HBE barrier function as TER may be governed by factors other than simply paracellular permeability to ions. It is worth noting that from day 7 to 8, when TER decreased from 800 to 700 Ohms × cm^2^, short circuit current increased from 6 to 8 μamps/cm^2^.

**Table 1:** Epithelial barrier parameters of differentiated 16HBE cell layers

	Potential difference (mV) (apical negative)	TER (Ω × cm^2^)	Short-circuit current (μA/cm^2^)	^14^C-Mannitol flux rate (pmol/min/cm^2^)
Mean ± SEM	−4.4 ± 0.2	713.7 ± 30	6.8 ± 0.4	2.1 ± 0.1
Range	−1.4 to −7.1	266–1524	2.3–15.8	1.5–4.0

Seeded at 1.0 × 10^6^ or 2.0 × 10^6^ cells per 4.2 cm^2^ PCF on day 0, potential difference, TER, short-circuit current, and ^14^C-mannitol flux rate were measured as described in ‘Materials and methods’ section on days 6, 7, or 8. The data include *n*=57 cell layers treated under control conditions in various experiments.

**Figure 3:**
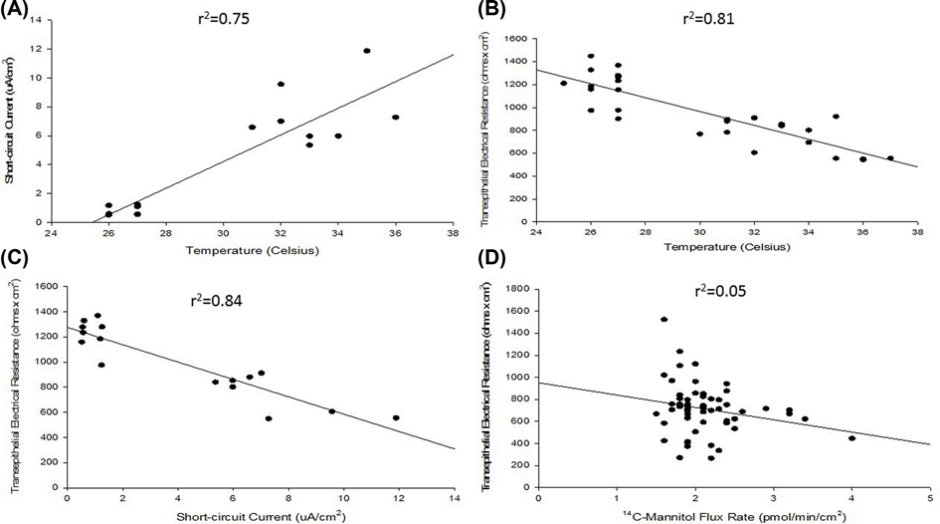
Relationships among barrier parameters for differentiated 16HBE cell layers (**A**) Short-circuit current dependence on temperature; (**B**) TER dependence on temperature; (**C**) TER dependence on short-circuit current; (**D**) lack of relationship between TER and ^14^C-mannitol flux rates for already formed 16HBE barriers. Seeded at 2.0 × 10^6^ cells per 4.2 cm^2^ PCF on day 0, TER and I_sc_ were determined as described in ‘Materials and methods’ section on day 7 post-seeding for *n*=8 cell layers (A,C) or *n*=16 cell layers (B) recorded at varying temperatures. For panel (D), *n*=57 cell layers treated under control conditions throughout various experiments were seeded at 1.0 × 10^6^ or 2.0 × 10^6^ cells per PCF on day 0, TER and J_m_ were determined as described in ‘Materials and methods’ section on days 6, 7, and 8 post-seeding.

It was hypothesized that perhaps the levels of CFTR channels expressed in differentiated 16HBE may enhance transcellular conductance of chloride to levels where it affects the overall TER. With TER values often approaching and exceeding 1000 Ω × cm^2^, this could be possible mathematically. As expected, we observed a strong temperature dependence for short-circuit current (I_sc_), a phenomenon that is very common in epithelial barrier models due to I_sc_’s ATP dependence (Figure 3A). However, it was observed that TER is heavily temperature dependent as well (Figure 3B). The two transepithelial parameters in fact have a strong inverse relationship (Figure 3C). At least some of the variability observed in TER values presented in Table 1 may be attributed to differences in differentiation state and/or temperature of the cell layer at the time of measurement, thereby influencing the I_sc_ value and in turn TER. This is a crucial consideration for any studies utilizing TER as an indication of 16HBE barrier function. The complex nature of the TER measurement for 16HBE cell layers is well illustrated in Figure 3D, where one can see cell layers with J_m_ values less than 2.0 pmol/min/cm^2^ featuring a range of TER values from 266 to 1524 Ω × cm^2^ with no clear (inverse) correlation between the two transepithelial barrier parameters (r^2^ = 0.05) as is usually seen for a variety of other epithelial cell culture models [25,26].

**Figure 4:**
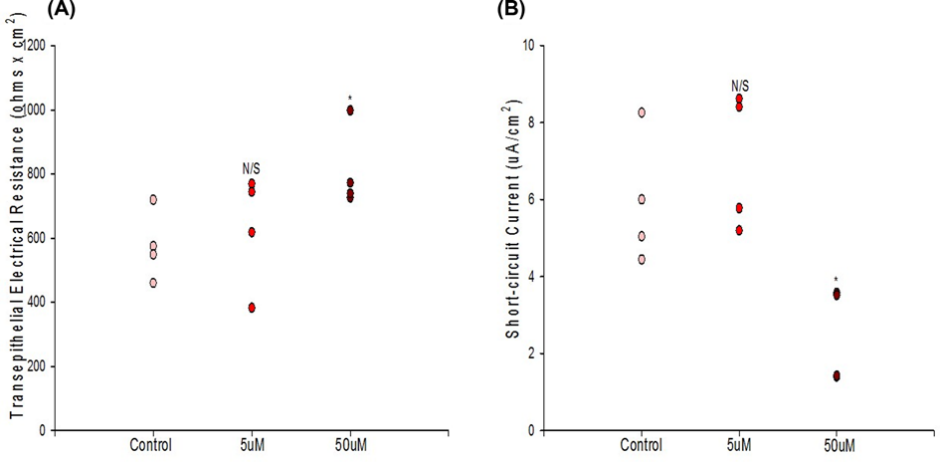
The effect of GlyH-101 on TER (A) and short-circuit current (B) Seeded at 2.0 × 10^6^ cells per PCF on day 0, treatment with appropriate concentration of GlyH-101 occurred on day 7 post-seeding and TER and I_sc_ were then measured 15 min post-treatment for *n*=4 cell layers per condition as described in ‘Materials and methods’ section. Bars represent mean ± standard error of the mean. * indicates *P*<0.05, N/S indicates not significant (Student’s *t* test, two-tailed).A

Pharmacologic inhibition of CFTR with 50 μM GlyH-101 significantly reduced I_sc_ compared with matched controls as expected, 15 min after treatment (Figure 4B). GlyH-101 was also observed to significantly increase TER in certain experiments (Figure 4A). No effect was observed on J_m_, suggesting that the effect on TER was mediated by the inhibition of chloride channels. Whereas the decrease in I_sc_ caused by GlyH-101 was always consistent in follow-up experiments, an effect on TER was sometimes not observed.

An apical-negative voltage of nearly 10 mV was observed when a 100 mM NaCl gradient was directed apically across a confluent cell layer, indicating that the TJs of 16HBE are anion-selective (Table 2). The P_Cl_/P_Na_ value (derived by the Goldman Equation) for 16HBE was 1.9 suggesting that the TJs are highly selective for chloride ions in these studies. Note that the saline solutions were supplemented with 10% fetal bovine serum to preserve barrier function throughout the experiments (TER_1_ vs. TER_2_), thus allowing for a more valid measurement of ion selectivity.

**Table 2:** Diffusion potential values across 16HBE cell layers

	ΔV_10_ (mV)	TER_1_ (Ohms × cm^2^)	TER_2_ (ohms × cm^2^)	P_Cl_/P_Na_
Mean ± SEM	−9.15 ± 0.4	780 ± 59	763 ± 57	1.91 ± 0.06

Diffusion potential measurements were performed as described in ‘Materials and methods’ section for *n*=4 cell layers. PCFs were seeded at 2.0 × 10^6^ cells at day 0 and the experiment was performed at day 7 post-seeding. ΔV_10_ represents the potential difference (apical negative) across 16HBE with an apically directed 100 mM NaC1 gradient at t = 10 min. TER_1_ represents the baseline TER value recorded in culture medium prior to the experiment and TER_2_ represents the TER value in Saline A after the experiment. P_Cl_/P_Na_ represents the relative permeability of the 16HBE cell layer to chloride vs sodium with the imposed 100 mM NaC1 gradient. (See Supplemental File regarding liquid junction potential measurements)

### Increased transepithelial barrier leak with TNF-α and TPA exposure

**Figure 5:**
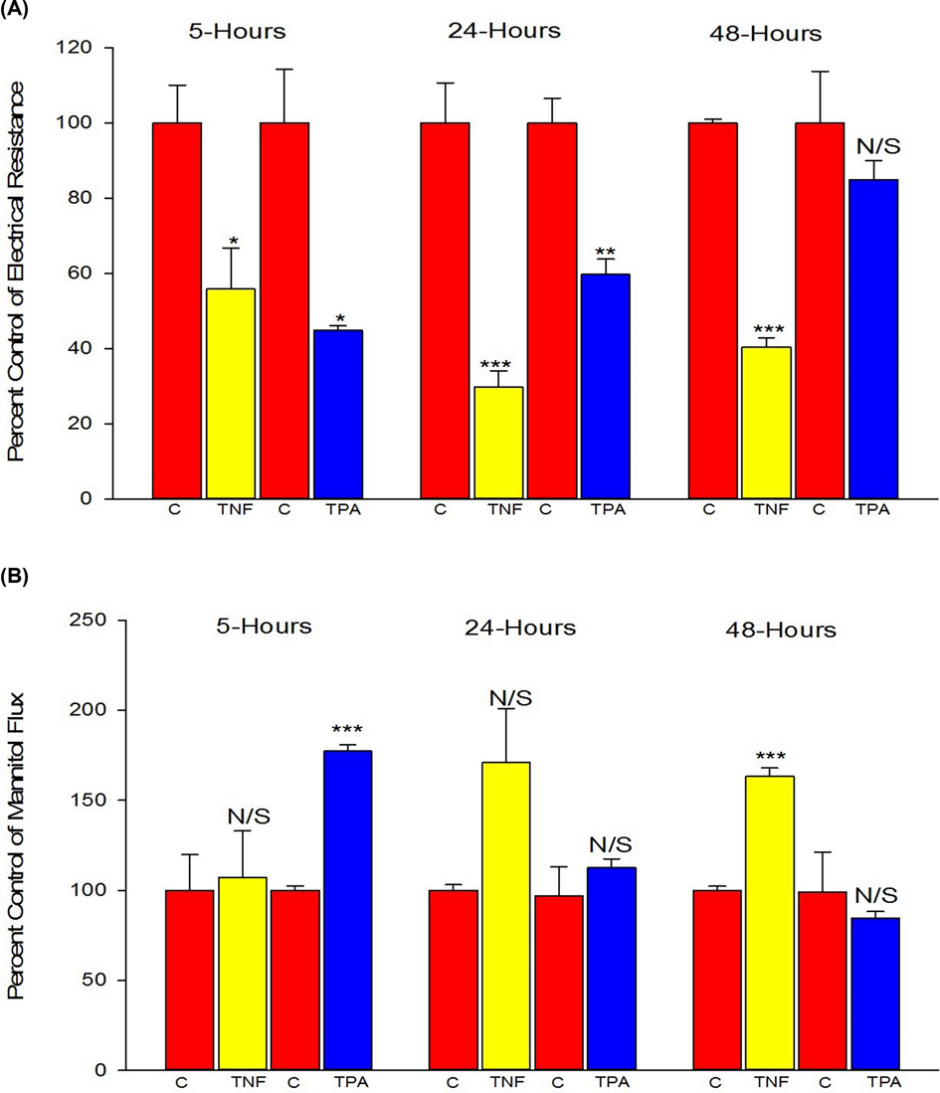
The effects of TNF-α or TPA on 16HBE TER (A) and ^14^C-mannitol flux rate (B) Cells were seeded on day 0 at 1.1 × 10^6^ cells per 4.2 cm^2^ PCF for the TNF-α experiments and at 1.2 × 10^6^ cells per PCF for the TPA experiments. Treatment with 133 ng/ml TNF-α or 10^−7^M TPA occurred on day 6 post-seeding as described in ‘Materials and methods’ section. At 5, 24, and 48 h post-treatment, TER and J_m_ measurements were recorded as described in ‘Materials and methods’ section. Data are represented as percent of control group within the same experiment ± standard error for *n*=3 cell layers for control and TPA groups at 5 h; *n*=4 for each condition for the two other time points. * indicates *P*<0.05, ** indicates *P*<0.01, *** indicates *P*<0.001, N/S indicates not significant (treatment compared with control for that experiment, Student’s *t* test, two-tailed). ‘C’ indicates control conditions.

The pro-inflammatory cytokine, TNF-α, and the phorbol ester, protein kinase C (PKC)-activator, TPA, were both effective in compromising 16HBE barrier function, but with very different time courses of action (Figure 5). TNF-α failed to dramatically affect barrier function compared with control until 24 h after the initial exposure, at which time a 71% increase in J_m_ and 70% decrease in TER were observed. TPA's effect on the other hand was much more rapid, being most effective at 5 h, causing a 77% increase in J_m_ and 55% decrease in TER. This effect of TPA dissipated over time even with re-exposure to fresh TPA at the 24-h time point suggesting down-regulation of PKC. Note, while TNF-α did significantly reduce TER at 5 h, there was no parallel increase in J_m._ Given the complexity of TER measurements outlined in the present paper, J_m_ was relied on more heavily when observing for changes in barrier strength.

**Figure 6:**
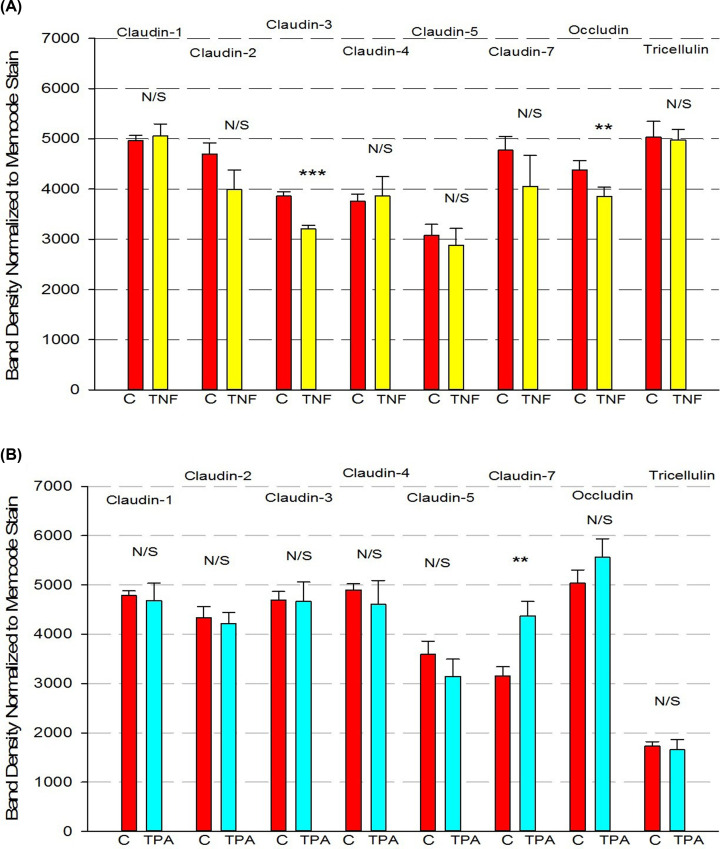
Effect of 24-h exposure to TNF-α (A) or TPA (B) on 16HBE tight junctional proteins Cell layers used in the barrier function studies from Figure 5 were harvested from filters, and the total cell lysates were analyzed for tight junctional proteins in Western immunoblots as described in ‘Materials and methods’ section. Data are represented as mean ± standard error for *n*=4 cell layers for each condition. ** indicates *P*<0.01, *** indicates *P*<0.001, N/S indicates not significant (treatment compared with control for that experiment, Student’s *t* test, two-tailed). ‘C’ indicates control conditions.

TNF-α significantly, though moderately, reduced expression of claudin-3 and occludin 24 h after exposure (Figure 6A), while TPA significantly elevated expression of claudin-7 (Figure 6B). Though the effects on 16HBE TJ proteins were moderate, these effects may highlight the sensitivity of barrier strength to any change in TJ proteins. No changes of claudins or occludin expression were observed 48 h after TNF or TPA exposure.

### Zinc and quercetin

**Figure 7:**
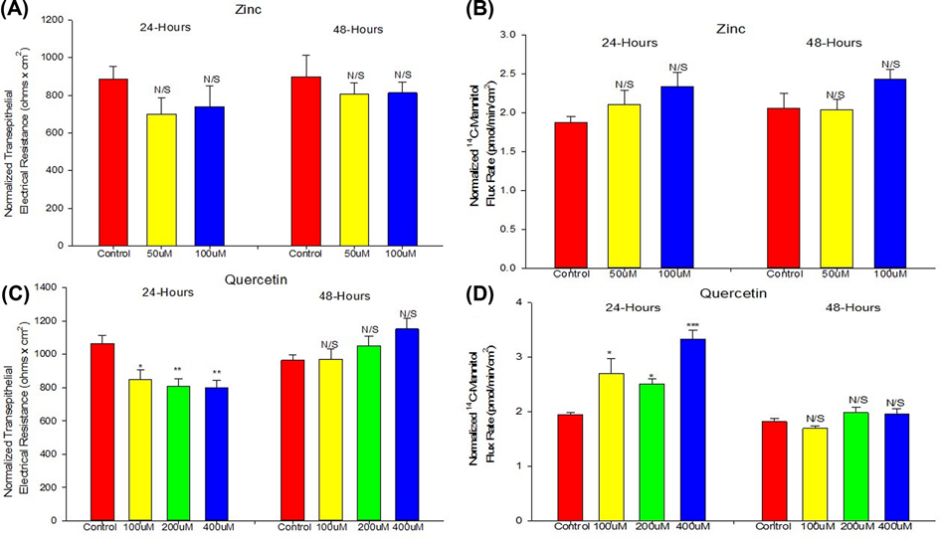
Effect of zinc or quercetin on 16HBE barrier function The effect of zinc on TER (**A**) and ^14^C-mannitol flux (**B**) as well as the effect of quercetin on TER (**C**) and ^14^C-mannitol flux (**D**) are shown. Cells were seeded on day 0 at 2.0 × 10^6^ cells per PCF. Treatment with the appropriate zinc or quercetin concentration occurred on day 6 as described in ‘Materials and methods’ section. At 24 and 48 h post-treatment, TER and J_m_ measurements were recorded as described in ‘Materials and methods’ section. Data are normalized mean ± standard error for *n*=8 cell layers. * indicates *P*<0.05, ** indicates *P*<0.01, *** indicates *P*<0.001, N/S indicates not significant (Holm–Sidak method following one-way ANOVA).

Although able to modify TJs and induce improved barrier function in various epithelial cell culture models, neither zinc nor quercetin were effective in enhancing 16HBE barrier function (Figure 7). Whereas quercetin at 400 μM did significantly increase TER at 48 h, this occurred with a significant decrease in I_sc_ and no corresponding decrease in J_m_. At 24 h, all concentrations of quercetin in fact exhibited true barrier leak (decreased TER and increased J_m_). At 24 h, 50 and 100 μM zinc showed no significant effect on TER but did increase mannitol leak. At 48 h, there was no significant effect on either. There was certainly no improvement in barrier function by either compound at any concentration.

## Discussion

The 16HBE, human bronchial epithelial cell line, has become a widely utilized model for a variety of respiratory epithelial physiological and disease processes [16–22]. 16HBE like Calu-3 are both airway-derived established epithelial cell lines, whose cells at confluence become polar with apical microvilli, form tight junctional seals and manifest cAMP-regulated CFTR (chloride) channels which are major contributors to the cell layers’ short-circuit current [29,30]. The 16HBE cell layers studied here developed TER values as high as 1000 Ohms × cm^2^, similar to the values seen with the Calu-3 airway epithelial cell line model [31,32] and normal bronchial epithelia in primary culture [33]. These values—obtained by culturing the cell layers under liquid (culture medium)—were found to be as high or higher than values obtained by air–liquid interface culture (approximately 300 Ohms × cm^2^) [31], although Mathia et al. (2002) [32] did report TER of 1000 Ohms × cm^2^ using air-interface culture. ZO-1 content was also higher when cultured under liquid. In addition to morphological and electrophysiological polarity, presence of apical CFTR chloride channels and presence of TJs, there are a wide range of other similarities among 16HBE, Calu-3, and primary bronchial epithelial cells, encompassing both structural proteins and regulatory pathways and proteins [34–38]. We would also point out that 16HBE does possess mucus-secreting capacity in part via the airway-specific mucin protein, MUC5AC, a further similarity to bronchial epithelia [39,33]. While 16HBE does lack cilia expression, it interestingly expresses the TRPV4 calcium channel thought to regulate cilia beating [40].

The issue of the legitimacy of established cell lines in epithelial cell physiological research has been contested by some for over 40 years, but the extremely wide use of these models in renal and gastrointestinal—as well as lung—studies seems to underscore their value. Over 500 papers were published using 16HBE in the last 10 years. The Calu-3 lung epithelial cell line has had over 500 as well. Despite the valid issue of precise relevance of established cell lines to the tissue of origin, there are undeniable advantages to using cell lines in biomedical research: homogeneity of cell type; ease of genetic manipulation; ability to continually return to the same cell population months or years after a study. Primary cell cultures certainly excel regarding relevance to the tissue of origin but can have an inherent disadvantage of heterogeneity of cell type (including even non-epithelial cells) as well as a pronounced phenotypic variability of the cell population over even short time periods as described by Becker and Willis (1979) [41].

Our current study provides information on the barrier function properties of 16HBE cell layers that would be useful to research groups utilizing this model. Confluent 16HBE cell layers’ intrinsic cell polarity and ability to form TJs [16] allow this model to form very robust barriers useful to a variety of studies and disease applications. The highlights of our current study with 16HBE cell layers are: (1) the cell layers post-confluence are able to form domes/hemicysts on solid surfaces, visual evidence of their polarity and barrier function (Figure 1B) [27,28]; (2) post-confluent cell layers are heterogeneously monolayered and multilayered (Figure 1C); (3) post-confluent cell layers on permeable supports display very robust barrier properties, with TER values typically ranging from 300 to 1500 Ohms × cm^2^, I_sc_ values from 2.3 to 15.8 μamps/cm^2^ and mannitol permeability that is quite low (1.5–4.0 pmoles/min/cm^2^ for a 0.1 mM mannitol reservoir) (Table 1); (4) diffusion potential studies show that 16HBE TJs are anion-selective, with a 1.9 P_C1_/P_Na_ ratio (Table 2); (5) the TJ proteins occludin, tricellulin, and claudins-1, -2, -3, -4, -5, and -7 are expressed (Figure 6); (6) the proinflammatory cytokine, TNF-α, and the phorbol ester, TPA, can both be used to induce stable leak across the cell layer, although such leak occurs with only moderate changes in only a few TJ protein expression levels (Figure 6); and (7) the commonly used barrier-enhancing micronutrients, zinc and quercetin [44–49], are surprisingly unable to improve barrier function across 16HBE cell layers (Figure 7).

The most important methodological finding of our study for researchers using the 16HBE cell line model is the relationship of TER to temperature, mannitol leak, and I_sc_. Whereas many epithelial cell culture models manifest a tight inverse correlation between TER and J_m_, the situation for 16HBE is complicated by the nature of its TER [42,43]. Although TER and J_m_ express an obvious inverse relationship as barrier function is developing (Figure 2), the tight relationship breaks down post-confluence once J_m_ values fall below 2 picomoles/min/cm^2^ (Figure 3D). This loss of relationship is not due to J_m_—which maintains a very narrow range post-confluence—but rather with TER, which exhibits much greater variability (Table 1). This variability (in measurement) of TER may relate to the surprisingly strong temperature dependence of TER values of 16HBE cell layers. Unlike the expected strong temperature dependence of I_sc_ (Figure 3A), TER also was observed to vary with temperature (Figure 3B). The observed (inverse) correlation between TER and I_sc_ (Figure 3C), highlights the fact that transcellular conductance may be playing a significant role in TER values in this model. It raises a warning to researchers to not simply rely on TER as a measure of this cell layer’s barrier function.

16HBE is known to possess strong CFTR channel activity post-confluence [16,17]. We confirm this in our own study by demonstrating the significant inhibitory action of the chloride channel inhibitor, GlyH-101, on I_sc_ (Figure 4B). GlyH-101’s site of action on the outside of the cell and its relatively high-water solubility make it preferential here to CFTR_inh_-172 [50,51]. As 16HBE cell layers are developing TJ (paracellular) resistance post-confluence, a significant transcellular conductive pathway is also developing. When one considers that the TER values for 16HBE cell layers are quite high, typically 1000 Ohms × cm^2^, and considering that this CFTR transcellular conductance lies in parallel with the paracellular conductance through TJs (where 1/R_T_ = 1/R_1_ + 1/R_2_), it is not surprising that we could observe an effect of CFTR inhibition on TER, i.e. the measured increase in TER in the presence of (apical) GlyH-101 (Figure 4A). Research groups using 16HBE cell layers in barrier function studies involving, e.g. cytokines or steroids, need to be aware that barrier function assessments focusing only on TER may not cleanly reflect changes in TJ/paracellular permeability, as would be true for many epithelial barrier models. Using paracellular probes such as ^14^C-d-mannitol, may provide a clearer reflection of TJ permeability changes in this model. For example, in our study, the significant increase in TER caused by 400 μM quercetin at 48 h is not reflected by a corresponding decrease in J_m_ (Figure 7C,D). Note that this increase in TER did coincide with a significant decrease in I_sc_.

Though inhibition of CFTR with GlyH-101 did not produce any change in paracellular permeability in our experiments, it is worth noting that Molenda et al. and Weiser et al. have reported an increase in paracellular flux of ^14^C-mannitol and fluorescein across 16HBE cell layers upon activation of CFTR with cAMP [52,53]. They suggested the following mechanism: CFTR activation increases chloride efflux and consequently depolarizes the plasma membrane, Rho/ROK activity increases as a result of the depolarization, and myosin light chain phosphorylation following Rho/ROK activation increases paracellular permeability. Given this mechanism, the difference in results obtained between our study and theirs’ regarding CFTR activity and paracellular permeability may be a function of inhibiting vs activating, namely differences in activity of the downstream pathways (Rho/ROK) when activating vs inhibiting CFTR. Inhibition of CFTR may not result in diminished Rho/ROK or MLCK activity (below basal level). Still, the fact that CFTR activation can alter barrier function by altering paracellular permeability in this cell line model is highly useful information. However, Nilsson et al. reported that activation or inhibition of CFTR in 16HBE cell layers reduced tight junctional leak [54]. These contradicting results highlight the complexity of CFTR’s role in regulating paracellular permeability. Note also that our own studies inhibiting CFTR activity by GlyH-101 were directed to a different goal, namely the complicating effect of CFTR activity on measurement of TER.

Forbes et al. and Wan et al. [23,55] observed 16HBE cell layers’ TER to peak between days 5 and 7 post-seeding (depending on initial seeding density) and then decline, consistent with the profile seen in our study (Figure 2) [23,55]. It should be noted that the culture conditions used in Forbes’ experiments differ from those used here in that Forbes et al. maintained 16HBE at an air–liquid interface. Apparently culturing 16HBE with both the apical and basal cell surfaces in contact with culture medium (as we did here) produces the same phenomenon for TER—namely a decline from peak values rather than a stable plateau. Overall, our data suggest that this TER decline is not due to an increasingly compromised barrier (see the stable J_m_ values after day 6 in Figure 2B) but rather increased transcellular conductance. It should be noted that air–liquid interface culturing is not essential for 16HBE differentiation/barrier formation, as two research groups have reported that conventional cell-culturing—as was done in our study—could produce stronger barrier function and better developed TJs in 16HBE cell layers [24,56].

Proinflammatory cytokines are often used as TJ-disrupting, leak-inducing agents. IFN-γ and IL-4, for example, have been shown to perturb TJ integrity and reduce TER in primary nasal epithelial cell layers [57]. Consistent with what we observed in Figure 5A, TNF-α has been shown to significantly compromise the barrier function of other epithelial barrier models such as CACO-2 and RPE cell layers in a time-dependent manner. The effect on 16HBE is not significant until 24 h after exposure and the effect is more pronounced at 48 h, similar to the time course of action that TNF-α has on CACO- 2 and RPE (Figure 5) [58,59]. In contrast, in LLC-PK_1_ cell layers, TNF-α has been shown to compromise barrier function as early as 90 min post-exposure [60]. TNF-α’s time course of action on 16HBE barrier function may indicate a transcriptionally regulated mechanism of action that could be explained by activation of the p38 MAPK pathway and subsequently elevated expression of non-muscle myosin light chain kinase [58–60]. It is worth noting TNF-α has been effective in inducing leak in primary human bronchial epithelial cells as well, though no changes in TJ proteins were observed via Western immunoblots [61]. In our study, small decreases in claudin-3 and occludin were observed in TNF-α-treated cell layers (Figure 6A).

Activation of various PKC isoforms through TPA has been shown to acutely and rapidly alter the phosphorylation state of TJ proteins and compromise barrier function of LLC-PK_1_ renal epithelium and ovarian cancer cell layers among others [62–64]. We observed a similar time course of action here for 16HBE cell layers, suggesting that the phosphorylation state of select TJ proteins may be important to the barrier strength of 16HBE cell layers as well. The pronounced changes in TER and J_m_ without corresponding pronounced changes in TJ protein expression levels (Figure 6B), suggests phosphorylation-based changes may be responsible. The fact that re-exposure to fresh TPA at the 24-h time point did not maintain the TPA-induced barrier compromise could likely be due to TPA-induced down-regulation of one or more classical PKC isoforms [44].

Zinc has been reported to enhance the basal barrier function of intestine and colon and has been implicated to play an important role in regulation of TJ protein expression in CACO-2 and Gie-3B11 cell culture models among others [45–47]. However, for 16HBE, zinc appears to have no positive effect at any of the concentrations used and may negatively alter barrier function above 100 μM (Figure 7A,B). Quercetin, like zinc, has been implicated in positively affecting gingival, renal, and intestinal epithelial barrier function and TJ protein expression [30–32]. With 16HBE, we observed only small negative effects, as 24-h exposure to all quercetin concentrations proved to be detrimental to 16HBE barrier function (Figure 7C,D). In this regard, it is worth noting that metabolites of quercetin have been counterintuitively shown to be prooxidative and negatively impact the male reproductive system in a manner that outweighs the antioxidant effects of quercetin [65,66].

In summary, in addition to providing general characteristics of the 16HBE cell line’s barrier morphology and transepithelial physiology–and describing the action of certain compounds known to affect barrier function in other epithelial models–we have demonstrated the complex nature of TER measurements across well-differentiated 16HBE cell layers, and recommend that research groups using 16HBE be aware of this complexity as well as the temperature and I_sc_ dependence of TER across 16HBE cell layers. This highlights the advisability of using paracellular probes, such as ^14^C-d-mannitol, for assessing barrier function in this model, and not relying solely on TER.

## Supplementary Material

supplemental File

## Abbreviations

CFTR: cystic fibrosis transmembrane conductance regulator
COPD: chronic obstructive pulmonary disease
HIV-1: human immunodeficiency virus 1
I_sc_: short-circuit current
J_m_: ^14^C-d-mannitol flux rate
LSC: liquid scintillation counting
PBS: phosphate buffered saline
PKC: protein kinase C
RT: room temperature
TER: transepithelial electrical resistance
TJ: tight junction
TNF-α: tumor necrosis factor-α
TPA: 12-*O*-tetradecanoylphorbol-13-acetate
16HBE: human bronchial epithelial cell line, 16HBE14o-
