# Supplementary material for: Epithelial barrier function properties of the 16HBE14o- human bronchial epithelial cell culture model
Source: Biosci Rep. 2020 Oct 16;40(10):BSR20201532. doi: 10.1042/BSR20201532 (PMC7569203; doi:10.1042/BSR20201532)
Supplement: supplemental File [file BSR-2020-1532_supp.pdf]

When we correct for the liquid junction potentials that would exist for our 1M NaCl agar bridges in contact with 150 mM NaCl vs 50 mM NaCl (osmotically balanced with mannitol) solutions (see schematic below), we observed that the diffusion potentials reported in Table 2 needed to be corrected up (more negative) by 3 mV, resulting in an adjusted P_{Cl}/P_{Na} ratio that is 2.5 rather than the originally reported 1.9. Correcting for existing liquid junction potentials thus does not change our original observation that this cell layer's junctional seals are anion selective.

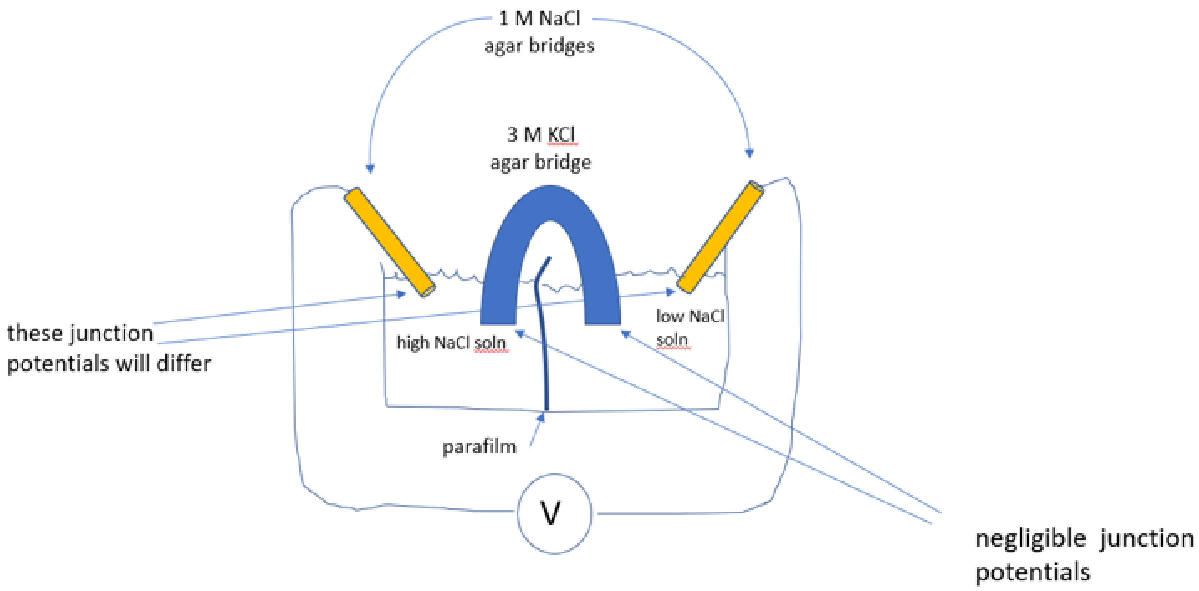

measured voltage will be asymmetry between the liquid junctions at the interfaces of test solutions
